# Immune Protection against Lethal Fungal-Bacterial Intra-Abdominal Infections

**DOI:** 10.1128/mBio.01472-17

**Published:** 2018-01-16

**Authors:** Elizabeth A. Lilly, Melanie Ikeh, Evelyn E. Nash, Paul L. Fidel, Mairi C. Noverr

**Affiliations:** a Center of Excellence in Oral and Craniofacial Biology, Louisiana State University Health Sciences Centre School of Dentistry, New Orleans, Louisiana, USA; b Department of Prosthodontics, Louisiana State University Health Sciences Centre School of Dentistry, New Orleans, Louisiana, USA; University of Texas Health Science Center

**Keywords:** *Candida albicans*, immune protection, *Staphylococcus aureus*, innate immunity, intra-abdominal infection

## Abstract

Polymicrobial intra-abdominal infections (IAIs) are clinically prevalent and cause significant morbidity and mortality, especially those involving fungi. Our laboratory developed a mouse model of IAI and demonstrated that intraperitoneal inoculation with *Candida albicans* or other virulent non-*albicans Candida* (NAC) species plus *Staphylococcus aureus* resulted in 70 to 80% mortality in 48 to 72 h due to robust local and systemic inflammation (sepsis). Surprisingly, inoculation with *Candida dubliniensis* or *Candida glabrata* with *S. aureus* resulted in minimal mortality, and rechallenge of these mice with lethal *C. albicans*/*S. aureus* (i.e., coninfection) resulted in >90% protection. The purpose of this study was to define requirements for *C. dubliniensis*/*S. aureus*-mediated protection and interrogate the mechanism of the protective response. Protection was conferred by *C. dubliniensis* alone or by killed *C. dubliniensis* plus live *S. aureus*. *S. aureus* alone was not protective, and killed *S. aureus* compromised *C. dubliniensis*-induced protection. *C. dubliniensis*/*S. aureus* also protected against lethal challenge by NAC plus *S. aureus* and could protect for a long-term duration (60 days between primary challenge and *C. albicans/S. aureus* rechallenge). Unexpectedly, mice deficient in T and B cells (Rag-1 knockouts [KO]) survived both the initial *C. dubliniensis/S. aureus* challenge and the *C. albicans/S. aureus* rechallenge, indicating that adaptive immunity did not play a role. Similarly, mice depleted of macrophages prior to rechallenge were also protected. In contrast, protection was associated with high numbers of Gr-1^hi^ polymorphonuclear leukocytes (PMNLs) in peritoneal lavage fluid within 4 h of rechallenge, and *in vivo* depletion of Gr-1^+^ cells prior to rechallenge abrogated protection. These results suggest that *Candida* species can induce protection against a lethal *C. albicans*/*S. aureus* IAI that is mediated by PMNLs and postulated to be a unique form of trained innate immunity.

## IMPORTANCE

Polymicrobial intra-abdominal infections are clinically devastating infections with high mortality rates, particularly those involving fungal pathogens, including *Candida* species. Even in patients receiving aggressive antimicrobial therapy, mortality rates remain unacceptably high. There are no available vaccines against IAI, which is complicated by the polymicrobial nature of the infection. IAI leads to lethal systemic inflammation (sepsis), which is difficult to target pharmacologically, as components of the inflammatory response are also needed to control the infection. Our studies demonstrate that prior inoculation with low-virulence *Candida* species provides strong protection against subsequent lethal infection with *C. albicans* and *S. aureus*. Surprisingly, protection is long-lived but not mediated by adaptive (specific) immunity. Instead, protection is dependent on cells of the innate immune system (nonspecific immunity) and provides protection against other virulent *Candida* species. This discovery implies that a form of trained innate immunity may be clinically effective against polymicrobial IAI.

## INTRODUCTION

Intra-abdominal infections (IAIs) are caused by the invasion and replication of microbes in the abdominal cavity (1, 2). Severe IAI can occur as a result of bowel perforation, laparotomy surgery, intestinal hernias, or insertion of medical devices, such as peritoneal catheters (3). If these infections are left untreated or misdiagnosed, microorganisms can migrate into the bloodstream, causing sepsis and leading to significant morbidity and mortality (4–6). IAIs are often polymicrobial, and infections involving both bacterial and fungal pathogens, such as *Candida albicans*, result in significantly higher mortality rates than infections involving bacterial species only (7–13). Bacterial coinfection during intra-abdominal candidiasis is common (up to 67%) (14). Along with Gram-negative enteric bacteria, Gram-positive species, including *Staphylococcus aureus*, are also frequently coisolated pathogens, particularly with nosocomial infections (15–20). Pathogenesis is not well understood, although inflammatory responses leading to sepsis are hypothesized to play a major role.

Our laboratory has been studying polymicrobial IAIs by using an experimental mouse model of *C. albicans*/*S. aureus* IAI (i.e., coinfection) which results in 70 to 80% mortality by 48 to 72 h postinoculation (21–23). Characterization of polymicrobial *C. albicans*/*S. aureus* IAI indicated that robust local and systemic inflammation is associated with mortality, as demonstrated by dramatically elevated levels of proinflammatory cytokines (interleukin-6, tumor necrosis factor alpha, and interleukin-1β) both locally and systemically despite a similar microbial burden and dissemination in monomicrobial infections (23). Treatment with indomethacin, a nonsteroidal anti-inflammatory drug (NSAID), prevented mortality, demonstrating a key role for inflammation in lethality (21).

One question arising from these studies was whether lethality was unique to *C. albicans* or whether other fungal species are also synergistically lethal with *S. aureus*. Subsequent studies using non-*albicans Candida* (NAC) species or non-*Candida* fungal species resulted in various levels of mortality. Coinfections with *S. aureus* plus *Candida glabrata* or *Saccharomyces cerevisiae*, both of which are avirulent in mouse models of systemic infection (22), resulted in no mortality. Coinfection with *S. aureus* plus *Candida krusei* or *Candida tropicalis* resulted in 80 to 90% mortality. However, *Candida dubliniensis*, a close phylogenetic relative of *C. albicans*, resulted in little to no mortality during coinfection, although infected mice showed some level of morbidity for a short time (22). In all cases, monomicrobial infections with NAC species, *S. cerevisiae*, or *S. aureus* alone were not lethal (22).

To investigate whether an avirulent coinfection could confer any protection against the lethal *C. albicans/S. aureus* coinfection, *C. dubliniensis/S. aureus-*infected mice were subsequently challenged 14 days after primary infection with a lethal dose of *C. albicans* plus *S. aureus*. Surprisingly, the *C. dubliniensis/S. aureus* primary challenge led to 70 to 80% protection (E. Nash and M. C. Noverr, unpublished results). The purpose of the present study was to define the requirements for inducing protective immunity and to identify the cellular mechanisms involved.

## RESULTS

### Requirements for protection against lethal polymicrobial IAI. (i) Role of NAC species.

**FIG 1:**
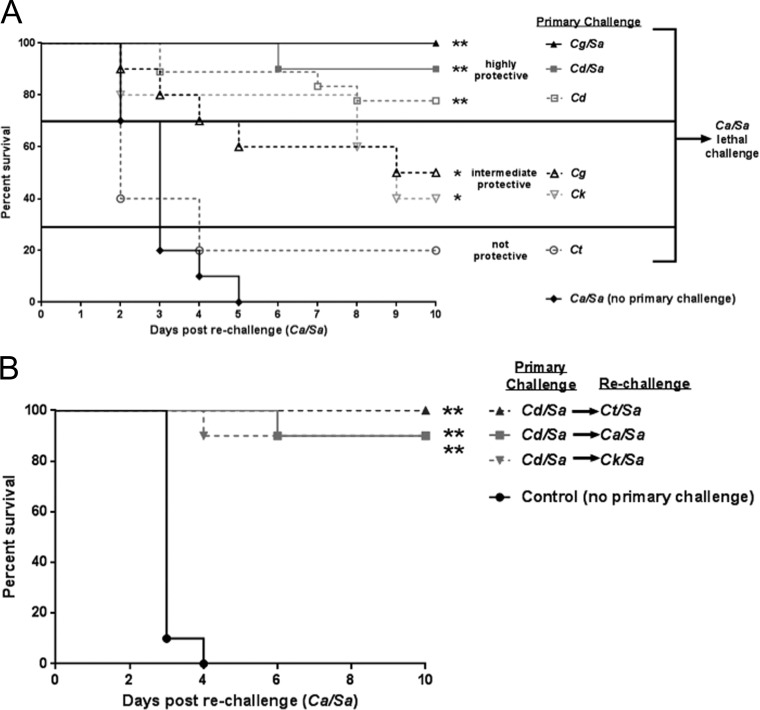
Role of NAC species in protection against lethal polymicrobial IAI. Mice (*n* = 10/group) were injected i.p. with 3.5 × 10^7^ CFU of *C. dubliniensis* (Cd) *C. glabrata* (Cg), *C. tropicalis* (Ct), or *C. krusei* (Ck) alone (standard inocula) or in combination with 8 × 10^7^ CFU of *S. aureus* (Sa) (standard inocula) as a primary challenge, and then rechallenged with *C. albicans/S. aureus* after 14 days (A) or injected i.p. with *C. dubliniensis/S. aureus* as the primary challenge and rechallenged with *C. albicans* (Ca), *C. tropicalis*, or *C. krusei* in combination with *S. aureus* after 14 days (standard inocula) (B). Animals receiving no primary challenge served as the positive (lethal) control. Mice were monitored for 10 days post-rechallenge. Data are representative of 2 separate experiments. *, *P* < 0.05; **, *P* < 0.0001 (significantly different from control by log rank Mantel-Cox test).

To build upon the initial observation that *C. dubliniensis/S. aureus* primary challenge confers protection against *C. albicans/S. aureus* coinfection, we sought to determine whether other NAC species could also confer protection with or without *S. aureus*. For this, groups of mice were inoculated with either a monomicrobial primary challenge of one of several NAC species (*C. krusei*, *C. tropicalis*, *C. dubliniensis*, or *C. glabrata*) or a polymicrobial challenge with *S. aureus*. Table 1 summarizes survival after the primary challenge. Monomicrobial infections resulted in 100% survival, consistent with previous reports (21, 22). Coinoculation with *C. dubliniensis/S. aureus* and *C. glabrata/S. aureus* resulted in 90 and 100% survival, respectively. *C. krusei*/*S. aureus* and *C. tropicalis*/*S. aureus*, on the other hand, showed reduced survival (50 and 40%, respectively), also consistent with previous reports, and animals were not rechallenged, in order to avoid survivor selection bias (22). All other groups were rechallenged after 14 days with a lethal inoculum of *C. albicans/S. aureus* and monitored for survival (Fig. 1A). A monomicrobial primary challenge with *C. krusei* or *C. glabrata* provided an intermediate level of protection upon rechallenge with *C. albicans*/*S. aureus* (40 to 50% survival) (*P* < 0.05). Primary challenge with *C. dubliniensis* conferred 80% protection (*P* < 0.0001). Primary challenge with *C. tropicalis* did not confer any level of protection. The addition of *S. aureus* in the primary challenge with either *C. glabrata* or *C. dubliniensis* increased survival following rechallenge (*C. glabrata*, 50%, and *C. glabrata*/*S. aureus*, 100%; *C. dubliniensis*, 80%, and *C. dubliniensis*/*S. aureus*, 90%).

**TABLE 1:** NAC species (with or without *S. aureus* coinfection) primary challenge survival

Primary challenge		% survival^*c*^ after primary challenge	MTD^*d*^ (days)
NAC species^*a*^	*S. aureus*^*b*^		
*C. dubliniensis*	−	100	NA
*C. glabrata*	−	100	NA
*C. krusei*	−	100	NA
*C. tropicalis*	−	100	NA
*C. dubliniensis*	+	90	3
*C. glabrata*	+	100	NA
*C. krusei*	+	50	4
*C. tropicalis*	+	40	6

a Inoculum of 1.75 × 10^7^ live *Candida* sp. cells injected i.p.

b Inoculum of 8 × 10^7^ live *S. aureus* cells injected i.p.

c Results are cumulative from 5 studies with a 14-day observation period.

d MTD, median time to death of mice that succumbed to infection. NA, not applicable.

Recognizing that coinfection with *C. krusei*/*S. aureus* or *C. tropicalis*/*S. aureus* results in ~50% mortality, we also tested whether primary challenge with *C. dubliniensis*/*S. aureus* conferred any level of protection against rechallenge with *C. krusei*/*S. aureus* or *C. tropicalis*/*S. aureus* (cross-protection). Interestingly, primary challenge with *C. dubliniensis*/*S. aureus* conferred 90 to 100% protection upon rechallenge with either *C. krusei/S. aureus* or *C. tropicalis/S. aureus* compared with naive mice (*P* < 0.0001) (Fig. 1B).

### (ii) Limits of *C. dubliniensis-*mediated protection.

**FIG 2:**
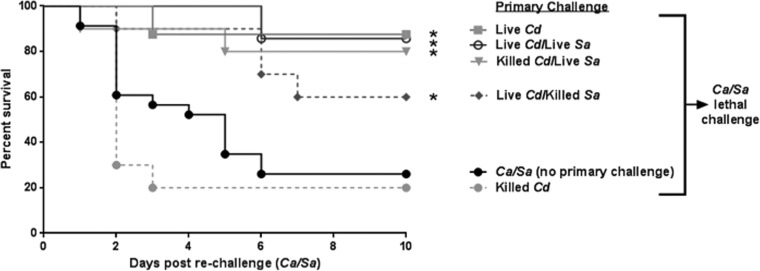
Limits of *C. dubliniensis-*mediated protection against lethal polymicrobial IAI. Mice (*n* = 10/group) were injected i.p. with different permutations of viable and nonviable *C. dubliniensis* and *S. aureus* as the primary challenge followed by rechallenge with *C. albicans/S. aureus* (standard inocula). Animals receiving no primary challenge served as the positive (lethal) control. Mice were monitored for 10 days post-rechallenge. Data are representative of 3 separate experiments. *, significantly different from control (*P* < 0.05) by log rank Mantel-Cox test.

We next sought to determine the requirements for protection by *C. dubliniensis* against *C. albicans/S. aureus* IAI. For this experiment, different permutations of viable and nonviable *C. dubliniensis* with or without *S. aureus* were given as the primary challenge, followed by rechallenge with *C. albicans/S. aureus*. Viable *C. dubliniensis* alone and nonviable *C. dubliniensis* plus live *S. aureus* both provided a high level of protection (80%) upon *C. albicans*/*S. aureus* rechallenge, compared to animals that received no primary challenge (*P* < 0.01) (Fig. 2). Interestingly, incorporating killed *S. aureus* into the primary challenge compromised live *C. dubliniensis*-induced protection by ~30%. Killed *C. dubliniensis* alone did not provide protection against subsequent *C. albicans/S. aureus* IAI (Fig. 2).

### (iii) Limits of *C. albicans-*mediated protection.

Because *C. dubliniensis*, a phylogenetically close relative of *C. albicans*, provided such a high level of protection alone against rechallenge with *C. albicans*/*S. aureus*, we tested whether *C. albicans* alone conferred similar protection. Similar to the *C. dubliniensis* studies, different permutations of viable and nonviable *C. albicans* and/or *S. aureus* were given as the primary challenge, followed by lethal *C. albicans*/*S. aureus* coinfection; animals were monitored for survival (Table 2). Surprisingly, monomicrobial primary challenge with live or killed *C. albicans* or *S. aureus* did not provide significant protection against *C. albicans*/*S. aureus* rechallenge. While a minority of mice (40%) given the initial *C. albicans* primary challenge survived, only 50% of the surviving mice were protected against the subsequent rechallenge with *C. albicans*/*S. aureus*. Reductions in the primary challenge inocula increased survival (to 90%) but failed to enhance protection beyond 70% (see Fig. S1A and B in the supplemental material). In contrast, primary challenge with killed *C. albicans* and live *S. aureus* resulted in 90% survival following rechallenge with *C. albicans*/*S. aureus*. The reverse combination (live *C. albicans*/killed *S. aureus*) failed to provide any level of protection, with a primary challenge mortality rate to similar to that with live *C. albicans* alone.

10.1128/mBio.01472-17.1 FIG S1  Limits of *C. albicans*-mediated protection. Mice (*n* = 10/group) were injected i.p. with the standard 2.5× inocula of viable *Ca* (1.75 × 10^7^), a 1× inocula (7 × 10^6^), or a 0.14× inocula of 1 × 10^6^ as the primary challenge followed by re-challenge with *Ca/Sa* (standard inocula) after 14 days. Animals receiving no primary challenge served as the positive (lethal) control. Mice were monitored for 10 days post re-challenge. Data are representative of 4 separate experiments. (A) Survival of animals in the 14-day primary challenge. (B) Survival of animals after re-challenge with *Ca/Sa*. Download FIG S1, PDF file, 0.1 MB. Copyright © 2018 Lilly et al. 2018 Lilly et al. This content is distributed under the terms of the Creative Commons Attribution 4.0 International license.

**TABLE 2:** Limits of *C. albicans*-mediated protection

Primary challenge		% survival after:	
*C. albicans*^*a*^	*S. aureus*^*b*^	Primary challenge^*c*^	Rechallenge with *C. albicans/S. aureus*
−	−	NA	25
Live *C. albicans*	−	40 (9)	50
Killed *C. albicans*	−	100	0
−	Live *S. aureus*	100	25
−	Killed *C. albicans*	100	20
Live *C. albicans*	Killed *S. aureus*	60 (5)	15
Killed *C. albicans*	Live *S. aureus*	100	90
Killed *C. albicans*	Killed *S. aureus*	100	0

a Inoculum of 1.75 × 10^7^ live or killed *C. albicans* was injected i.p.

b Inoculum of 8 × 10^7^ live or killed *S. aureus* was injected i.p.

c Results are cumulative from 6 studies with a 14-day observation period. NA, not applicable. Values in parentheses indicate the median time to death of mice that succumbed to infection.

### (iv) *C. dubliniensis* induces long-term protection against polymicrobial IAI.

**FIG 3:**
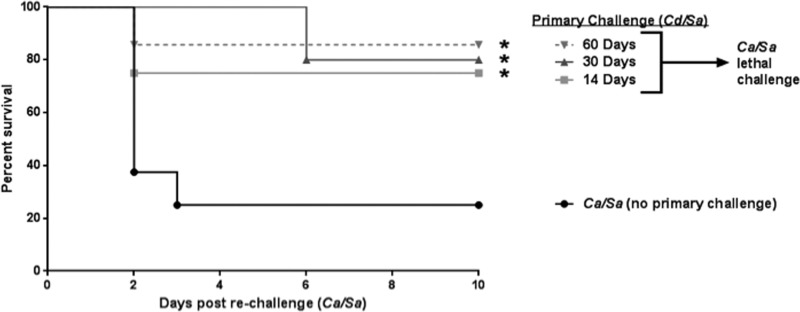
*C. dubliniensis* induces long term protection against polymicrobial IAI. Mice (*n* = 10/group) were injected i.p. with *C. dubliniensis* and *S. aureus* as the primary challenge 14, 30, and 60 days prior to rechallenge with *C. albicans / S. aureus* (standard inocula). Animals receiving no primary challenge served as the positive (lethal) control. Mice were monitored for 10 days post-rechallenge. *, significantly different from control (*P* < 0.05) by log rank Mantel-Cox test.

To determine if the protection conferred by *C. dubliniensis*/*S. aureus* would extend beyond the 14-day postchallenge period, mice were rechallenged with lethal *C. albicans*/*S. aureus* either 30 or 60 days following primary challenge. Consistent with previous results, at 14 days high-level protection (75 to 90% survival) (*P* < 0.05) was observed in animals rechallenged up to 60 days after the primary challenge compared to naive animals (Fig. 3).

### Mechanisms involved in *C. dubliniensis*-induced protection. (i) Role of T and B cells.

**FIG 4:**
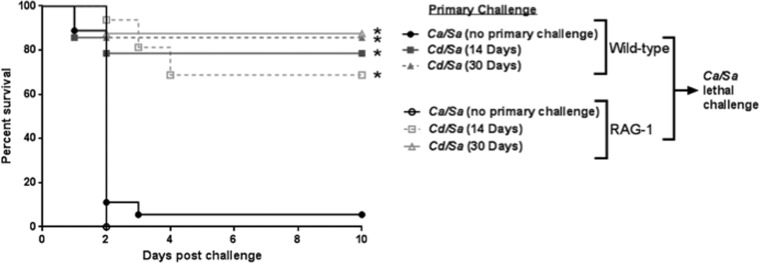
Role of T and B cells in *C. dubliniensis*-induced protection. RAG mice (deficient in T and B cells) (*n* = 10) and the background congenic strain, C57BL/6J mice (*n* = 10) were given the primary challenge of *C. dubliniensis* and *S. aureus* 30 days or 14 days prior to rechallenge with *C. albicans/S. aureus* (standard inocula). Animals receiving no primary challenge served as the positive (lethal) controls. Mice were monitored for 10 days post-rechallenge. *, significantly different from control (*P* < 0.05) by log rank Mantel-Cox test.

To determine the role of adaptive immunity in mediating protection against lethal IAI, *rag-1* knockout (KO) mice, which lack T and B cells, were used in primary and secondary challenge experiments. Both wild-type (C57BL/6J) and *rag-1* KO mice survived primary challenge with *C. dubliniensis*/*S. aureus* (data not shown). Unexpectedly, high-level protection was observed in both wild-type (80 to 90% survival) and *rag-1* KO mice (70 to 90% survival; *P* < 0.001) following lethal rechallenge with *C. albicans*/*S. aureus*, compared with naive mice, which succumbed to the lethal challenge within 2 days (Fig. 4).

### (ii) Role of macrophages.

**FIG 5:**
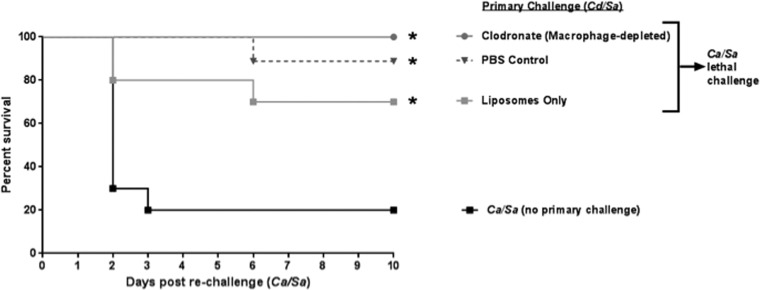
Role of macrophages in *C. dubliniensis*-induced protection. Mice (*n* = 10/group) previously given the primary challenge of *C. dubliniensis*/*S. aureus* (14 days prior) were injected i.p. with liposome-encapsulated clodronate (which results in ~90% depletion of resident peritoneal macrophages), liposomes only, or PBS 1 day prior to rechallenge with *C. albicans/S. aureus*. Animals receiving no primary challenge also served as the positive (lethal) controls. Mice were monitored for 10 days post-rechallenge. *, significantly different from control (*P* < 0.02) by log rank Mantel-Cox test.

To evaluate the role of resident peritoneal macrophages in mediating protection in *C. dubliniensis*/*S. aureus*-infected mice, liposome-encapsulated clodronate was injected intraperitoneally (i.p.) 1 day prior to rechallenge with *C. albicans*/*S. aureus*, which resulted in ~90% depletion of peritoneal macrophages (Fig. S2A). Empty liposomes or phosphate-buffered saline (PBS) alone were administered to control animals. All treated animals given the primary *C. dubliniensis*/*S. aureus* challenge showed high-level protection (75 to 100%) upon rechallenge compared to the control group, which received no primary challenge (*P* < 0.02) (Fig. 5).

10.1128/mBio.01472-17.2 FIG S2  Confirmation of macrophage and PMNL depletion. (A) *Cd/Sa* mice (*n* = 10/group) were injected i.p. with liposome-encapsulated clodronate one day prior to re-challenge with *Ca/Sa* to deplete macrophages. Empty liposomes or PBS alone were administered to control animals. Peritoneal lavage fluid was analyzed by flow cytometry to confirm depletion with red arrows indicating F4/80^+^ cells (macrophages). (B) Mice (*n* = 10/group) given the primary challenge of *Cd/Sa* were injected i.p. with 200 μg anti-Gr-1 (Ly6G/C) antibodies to deplete PMNLs or isotype control antibodies 48 h prior to and 2 h after re-challenge with *Ca/Sa*. Peritoneal lavage fluid was analyzed by flow cytometry to confirm depletion just prior to re-challenge with *Ca/Sa*. PMNLs are shown within the red encircled areas. Download FIG S2, PDF file, 0.1 MB. Copyright © 2018 Lilly et al. 2018 Lilly et al. This content is distributed under the terms of the Creative Commons Attribution 4.0 International license.

### (iii) Role of polymorphonuclear leukocytes

**FIG 6:**
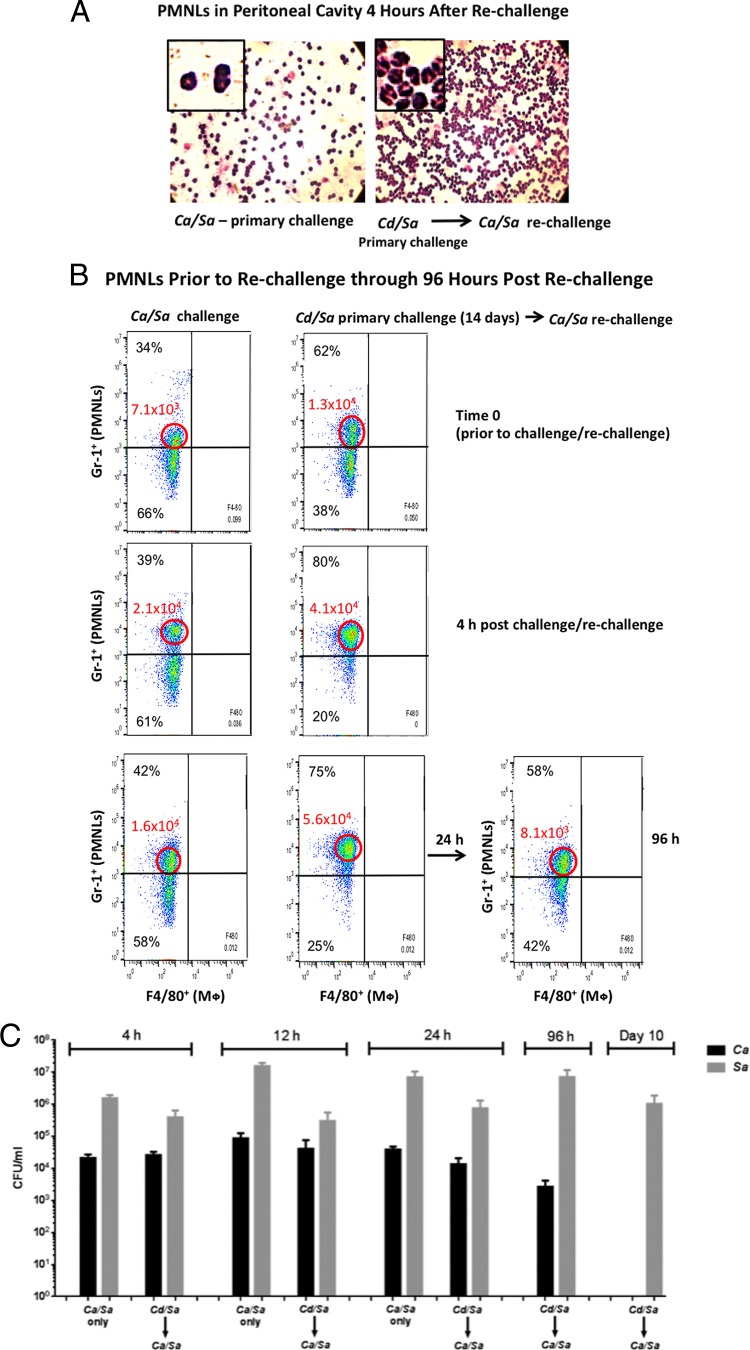
Presence of PMNLs in *C. albicans/S. aureus* rechallenged, protected animals. Mice (*n* = 10/group) were given the primary challenge of *C. dubliniensis/S. aureus* and rechallenged with *C. albicans*/*S. aureus* 14 days later. Control mice (*n* = 10) received no primary challenge. (A) H&E-stained smears of PMNLs from peritoneal lavage fluid collected 4 h after rechallenge. The illustration is representative of several individual mice evaluated. (B) Flow cytometry analysis results of PMNLs from peritoneal lavage fluid prior to rechallenge through 96 h post-rechallenge with *C. albicans/S. aureus*. Percentages indicate proportions of Gr-1^hi^ PMNLs present in the total cell population. MFI of Gr-1^hi^ PMNLs within the encircled areas are shown in red. The illustration is representative of results for several individual mice evaluated. (C) Microbial burden (*C. albicans* and *S. aureus*) in peritoneal lavage fluid of mice 4 h post-rechallenge through 10 days post-rechallenge with *C. albicans/S. aureus* in those that remained alive. Data are cumulative for all animals from each group. Mφ, macrophage(s).

Our previous studies demonstrated that both lethal polymicrobial and nonlethal monomicrobial primary infections are associated with a significant influx of polymorphonuclear leukocytes (PMNLs) into the peritoneal cavity. Therefore, we investigated whether similar PMNL recruitment occurs following *C. albicans*/*S. aureus* rechallenge of *C. dubliniensis*/*S. aureus-*infected animals. Hematoxylin and eosin (H&E) staining of peritoneal lavage fluid showed substantially higher PMNL levels in the peritoneal cavity, as early as 4 h after rechallenge, compared to control animals given the lethal challenge alone (Fig. 6A). These observations were confirmed and extended quantitatively by flow cytometry using Gr-1 antibody, which recognizes both Ly6G and Ly6C markers (Fig. 6B). Not only were Gr-1^hi^ cells present in the peritoneal lavage fluid at ~2-fold-higher levels in the rechallenged animals than in naive challenged mice from the time of inoculation (time zero) through 24 h postinoculation, but also the median fluorescence intensity (MFI) of the Gr-1^hi^ cells was 2- to 3-fold higher in rechallenged mice than in the naive challenged mice through the 24-h period. In rechallenged mice, the MFI of the Gr-1^hi^ cells increased 3- to 4-fold over the 24-h period, whereas a similar increase in the MFI was observed at 4 h in naive challenged mice and was followed by a reduction at 24 h. By 96 h in surviving rechallenged mice, Gr-1^hi^ cells returned to baseline levels, similar to levels in naive mice at time zero. Analysis of microbial burdens in peritoneal lavage fluid from animals at 4, 12, and 24 h post-lethal challenge showed considerable levels of both *S. aureus* (10^5^ to 10^6^ CFU/ml) and *C. albicans* (10^4^ to 10^5^ CFU/ml) in both primary infection and rechallenged mice, with no significant differences between the groups at early time points. By 96 h postchallenge, *S. aureus* remained high in the protected rechallenged animals, while *C. albicans* was reduced ~1 log (all naive primary challenged animals had succumbed to the infection). At the end of the observation period (10 days), *S. aureus* CFU remained high in the protected rechallenged mice (90% of which were still alive), while *C. albicans* CFU had been cleared (Fig. 6C).

**FIG 7:**
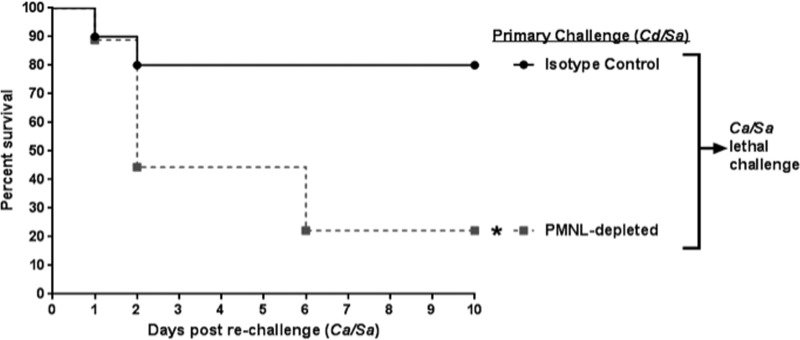
PMNL depletion abrogates protection. Mice (*n* = 10/group) given the primary challenge of *C. dubliniensis*/*S. aureus* were injected i.p. with 200 µg anti-Gr-1 (Ly6G/C) antibodies to deplete PMNLs or isotype control antibodies 48 h prior to and 2 h after rechallenge with *C. albicans/S. aureus*. Antibodies were given every 2 days to the remaining live animals for the duration of the study. Mice were monitored for 10 days post-rechallenge. *, significantly different from control (*P* = 0.02) by log rank Mantel-Cox test.

To confirm a role for PMNLs in protection, mice inoculated with the primary challenge of *C. dubliniensis*/*S. aureus* were injected i.p. with anti-Gr-1 antibodies to deplete PMNLs, or with isotype control antibodies, 48 h prior to and 2 h after rechallenge with *C. albicans*/*S. aureus*. Antibodies were given every 2 days thereafter to the remaining live animals for the duration of the study (10 days). As confirmation of PMNL depletion, flow cytometry analysis of peritoneal lavage fluid just prior to rechallenge with *C. albicans*/*S. aureus* showed ~60% reduction of Gr-1^hi^ PMNLs (of animals that received a single injection of anti-Gr-1 antibody), in comparison with control animals that were administered isotype antibodies (Fig. S2B). Significantly reduced survival (20%) was observed in mice, with an ~60% reduction in PMNLs upon rechallenge with *C. albicans*/*S. aureus*, similar to negative-control animals who did not receive the primary challenge (naive mice) (Fig. 7). Positive-control animals receiving the isotype antibodies showed significant protection, with 80% survival (*P* = 0.02).

## DISCUSSION

Our previous studies demonstrated wide variability in the ability of NAC species to induce synergistic lethality with *S. aureus* during polymicrobial IAI. An interesting follow-up study demonstrated that mice that survived a relatively avirulent polymicrobial challenge of *C. dubliniensis* and *S. aureus* exhibited high-level protection (80 to 90% survival) against a lethal *C. albicans*/*S. aureus* challenge. Here, we showed that protection was observed in rechallenged animals given the *C. dubliniensis*/*S. aureus* primary challenge up to 60 days prior to lethal challenge. While the long-term protection was suggestive of a role for adaptive immunity, protection was similarly observed in *rag-1* KO mice, which are deficient in both T and B cells, suggesting a possible role for innate immunity. While immunologic memory is a key feature of adaptive immunity, more recently the term “trained innate immunity” has been used to describe innate immune cells, primarily monocytes and macrophages, that exhibit enhanced responsiveness upon reinfection (24). This memory-like phenotype is mediated by epigenetic modifications and metabolic changes after initial pathogen exposure, resulting in a reprogramed or trained innate immune cells (24) capable of responding more productively to a secondary exposure. However, the high level of protection that remained in macrophage-depleted animals compared to PMNL-depleted animals reduced the likelihood for a role for macrophage-mediated trained innate immunity and instead suggested a role for PMNL-mediated trained innate immunity.

The presence of visibly increased numbers of PMNLs in peritoneal lavage samples as early as 4 h post-lethal challenge in rechallenged mice compared to naive challenged mice was the first evidence that suggested a role for PMNLs in protection. Further analysis by flow cytometry revealed a distinct population of Gr-1^hi^ PMNLs present in *C. dubliniensis*/*S. aureus* primary challenged mice at the time of lethal rechallenge, with increasing intensity of Gr-1^hi^ over the following the 24 h. While a similar Gr-1^+^ cell population was observed and was increased in naive control animals that received the lethal challenge only, the cell-associated intensity of Gr-1^hi^ cells never reached that observed in protected rechallenged mice. In addition, the Gr-1 intensity continued to decline at 24 h postinoculation in naive challenged mice. These results suggested that the presence of the Gr-1^hi^ cells in the peritoneal cavity at the time of challenge is important for protection and that further migration of Gr-1^hi^ PMNLs over a 24-h period promotes survival.

Protection by PMNLs was confirmed using antibody depletion (anti-Ly6G/C) with antibody-treated mice that exhibited significantly reduced survival compared to isotype-treated mice (~20% versus ~80% survival). Interestingly, while Gr-1^+^ cellular depletion was not 100% effective (~60%), the reduction was clearly sufficient during the 24-h period post-lethal challenge to reduce/eliminate protection. Incomplete antibody-mediated depletion of Gr-1^+^ cells has been previously reported, and it is possibly due to the presence of resistant cells residing in tissues (25). Interestingly, peritoneal microbial burden was very similar in both naive and protected mice challenged with lethal *C. albicans*/*S. aureus* coinfection at 4, 12, and 24 h postchallenge. This indicates that the protective response may act by controlling lethal inflammation rather than promoting antimicrobial activities. This is in agreement with our previous studies showing significant protection during IAI with NSAID treatment (21).

We hypothesize that the trained innate immunity conferred by Gr-1^+^ cells acts to reduce or control local and/or systemic inflammation to sublethal or subseptic levels. This controlled inflammatory response eventually leads to fungal clearance by day 10 postchallenge, and the residual bacterial burden is tolerated. Fungal clearance may be mediated by the same PMNLs or, alternatively, by other Gr-1^+^ PMNLs. It is unclear why *S. aureus* CFU counts remained high throughout the infection until it was eventually cleared. In addition, *S. aureus* is often still detected in animals given a primary challenge at the time of rechallenge (14 days), albeit at considerably lower levels (data not shown). Future studies can address these interesting monomicrobial conditions as well as the anti-*Candida* response in protected animals.

This PMNL-mediated protection is the first report of trained innate immunity mediated by Gr-1^+^ cells. Moreover, the protection we demonstrated was long-lived (up to 60 days postchallenge). This is particularly surprising, considering that the major population of Gr-1^+^ PMNLs are neutrophils, which are short-lived cells. A major question then arises: are these Gr-1^+^ PMNLs neutrophils or another type of polymorphonuclear leucocyte? Interestingly, myeloid-derived suppressor cells (MDSCs) are phenotypically similar to neutrophils (they are Gr-1^+^ and exhibit a polymorphonuclear granulocytic phenotype) with a similar lineage, arising from myeloid precursors in the bone marrow (26, 27). Known for their immunosuppressive properties, MDSCs infiltrate cancer tissues to regulate other immune cells, and they are much longer lived than neutrophils (26–28). High levels of MDSCs at these sites are associated with poor patient prognosis, making them a key therapeutic target for cancer treatment (28). In relation to our model, one may postulate that the inflammatory insult (infection) in the peritoneal cavity results in mobilization and expansion of MDSCs in the bone marrow.

Recruited MDSCs may act by reducing or controlling the inflammatory response, which prevents lethal sepsis. It is known that MDSCs exert direct antimicrobial activity, including activity against *Candida* species, and they may also directly participate in reductions in the microbial burden in protected animals (29, 30). In support of this hypothesis, murine MDSCs, which are heterogeneous, express high levels of Gr-1 (Ly6G and/or Ly6C), and depletion of these populations via an anti-Gr-1 antibody abrogated protection in our model. Initial attempts at depletion using only antibodies against Ly6G, which is predominantly expressed on neutrophils and subsets of MDSCs, failed to abrogate protection, possibly due to an inability to target all MDSC populations (data not shown). Further studies will interrogate the role for MDSC subsets as trained innate immune cell populations involved in protection against *C. albicans*/*S. aureus* IAI.

We also tested the ability of several other NAC species in a primary challenge with or without *S. aureus* to induce protection against lethal *C. albicans*/*S. aureus* rechallenge. Interestingly, a monomicrobial primary challenge of *C. krusei* or *C. glabrata* provided an intermediate level of protection, along with *C. krusei*/*S. aureus* and *C. tropicalis*/*S. aureus* coinfections, while *C. glabrata*/*S. aureus* and *C. dubliniensis*/*S. aureus* coinfections conferred the strongest protection. Like the lethal outcome from *Candida*/*S. aureus* challenge (21–23), protection against *C. albicans*/*S. aureus* lethal challenge was species specific and unrelated to morphology. *C. glabrata* only grows in the yeast form, and *C. dubliniensis* grows as both yeast and hyphae. Taking into account these results, we chose to focus specifically on *C. dubliniensis* due to the fact it is a close phylogenetic relative of *C. albicans* but exhibits low virulence in most animal models and is relatively rare clinically as an etiologic agent of infection (31–33). Although *C. glabrata* also exhibits low virulence in animal models (34, 35) and provided protection in our model, *C. glabrata* was not a good candidate for further study because it is a common etiologic pathogen in clinical situations and exhibits considerable innate antifungal resistance (36, 37). Furthermore, *C. dubliniensis* conferred high-level protection even in the absence of *S. aureus*, whereas *S. aureus* was required with *C. glabrata* to confer a similar level of protection, providing further support for our focus on *C. dubliniensis*.

In subsequent studies, killed *C. dubliniensis*/live *S. aureus* and live *C. dubliniensis*/killed *S. aureus* also conferred protection, but killed *C. dubliniensis*/killed *S. aureus* was not protective (data not shown). Of note, although live *C. dubliniensis*/live *S. aureus-*challenged mice exhibited 90% survival (Table 1), all showed initial signs of morbidity, as previously reported (22), with low mortality (10%), usually within 72 h postinoculation. This was unchanged even at 5-fold-higher inoculum levels (data not shown). Overall, these results suggest that several species of *Candida* can induce protection against the lethal *C. albicans*/*S. aureus* challenge, that *S. aureus* is usually required, and at least one of the two organisms must be viable. It is interesting that *C. dubliniensis* can induce high-level protection in the absence of *S. aureus* (and with no signs of morbidity). This may be due to the genetic relatedness of *C. dubliniensis* and *C. albicans*, such that the initial interactions with host cells mimic *C. albicans* but with reduced virulence or host damage. The genetic similarities and ability to induce cross-protection against other NAC species raise the question of whether *C. dubliniensis* vaccination also induces antigen-specific responses against proteins common to all *Candida* species. For example, the *C. albicans* Als3 vaccine is protective against intravenous infection with either *C. albicans* or *S. aureus*, due to antigenic similarity with a bacterial surface adhesin called clumping factor (38, 39). It is tempting to speculate that *C. dubliniensis* induces a similar response; however, the *ALS3* gene is absent in *C. dubliniensis* and could not support induction of antigen-specific responses against the protein, so the mechanisms involved are clearly distinct. Equally interesting is that *C. dubliniensis* can provide protection against *C. tropicalis*/*S. aureus* and *C. krusei*/*S. aureus* coinfections, which are otherwise as lethal as *C. albicans*/*S. aureus* coinfection. Hence, the protection has a broad-spectrum nature toward several *Candida* species.

Recognizing the efficacy of protection provided by *C. dubliniensis*, it was surprising that *C. albicans* alone (monomicrobial challenge) could not confer a similar level of protection. However, the combination of killed *C. albicans*/live *S. aureus* primary challenge conferred a high level of protection similar to that of killed *C. dubliniensis/*live *S. aureus* primary challenge. A possible reason for the lack of protection from a live *C. albicans* primary challenge is that it creates considerable damage in the host, even in the monomicrobial setting, that is difficult to overcome even if a protective response is generated. Of note, the monomicrobial live *C. albicans*-challenged mice given the standard inocula had an ~60% mortality rate, which was inconsistent with our previous report for monomicrobial infection (21). However, the previous report entailed outcomes at 5 days postinfection. The mice in this study had a median time to death of 9 days. Yet, even with a 2.5-fold and 17.5-fold reduction in the monomicrobial challenge that increased survival to 70 to 90%, protection was still modest compared to that from a *C. dubliniensis* primary challenge (50 to 70%). Hence, *C. dubliniensis* is clearly superior to any other *Candida* species for induction of protection.

As for the role of *S. aureus* in protection, viable *S. aureus* is required when killed *C. dubliniensis* or *C. albicans* is given in the primary challenge. The addition of *S. aureus* in the primary challenge can also moderately enhance protection when given with live *C. glabrata* or *C. dubliniensis*, but with no ability to induce the protective response alone. Interestingly, inclusion of killed *S. aureus* with live *Candida* (*C. dubliniensis* or *C. albicans*) compromised any protection provided, suggestive of an enhanced inflammatory response in the host from killed *S. aureus* components, which in turn promoted sepsis, presumably independently, that dampened the protective effects of *Candida*. This phenomenon is similar to another model of lethal fungal IAI that entails live *C. albicans* with sterile feces (including killed bacteria) (40). Together, these results suggest that the *Candida* component of the primary challenge is the key driving force behind the protective response and that *S. aureus* is more or less a contributing factor or provides an adjuvant-like effect.

In summary, *C. dubliniensis* is a viable vaccine candidate or therapeutic agent to protect against lethal polymicrobial IAI involving virulent *Candida* species. This protection is mediated by a specific population of Gr-1^+^ PMNLs that are phenotypically similar to neutrophils but that could potentially be MDSCs, and these cells provide long-lived protection by a trained innate immune mechanism never before reported. Future studies will be focused on prospects of using *C. dubliniensis* as a vaccine candidate and characterizing the specific pathways and training mechanisms involved in induction of the protective PMNLs and the subsequent effector mechanisms that are required for mediating protection against lethal polymicrobial IAI.

## MATERIALS AND METHODS

### Mice.

For most experiments, female Swiss Webster mice, 5 to 7 weeks of age, were purchased from Charles River Laboratories, Inc. Additional studies used female RAG-1 KO and C57BL/6J mice (Jackson Laboratories). Animals were housed and handled according to institutionally recommended guidelines. All experiments involving animals were approved by the Louisiana State University Health Sciences Centre (LSUHSC) Institutional Animal Care and Use Committee.

### Strains and growth conditions.

*C. albicans* strain DAY185, a prototrophic derivative of SC5314, was a gift from Aaron Mitchell (Carnegie Mellon University, Pittsburgh, PA). All other *Candida* species, with the exception of *C. krusei*, were provided by Jack Sobel (Wayne State University, Detroit, MI). *C. krusei* was obtained from the Fidel laboratory bank of isolates (LSUHSC, New Orleans, LA.) Frozen stocks were maintained at −80°C and streaked onto yeast extract-peptone-dextrose (YPD) agar prior to use. A single colony was transferred to 10 ml of YPD broth and the culture was shaken at 30°C for 12 to 18 h. The methicillin-resistant *S. aureus* strain NRS383 used in all experiments was obtained from the Network on Antimicrobial Resistance in *Staphylococcus aureus* (NARSA) data bank. Frozen stocks were maintained at −80°C and streaked onto Trypticase soy agar (TSA) prior to use. A single colony was transferred to 10 ml of Trypticase soy broth (TSB) and shaken at 37°C overnight. On the following day, the overnight culture was diluted 1:100 in fresh TSB and shaken at 37°C for 3 h until the culture reached the log phase of growth. Prior to inoculation, cultures of both organisms were washed 3 times by centrifugation in sterile PBS (pH 7.4), counted using a hemocytometer, and diluted in sterile PBS to prepare standardized inocula. For experiments using UV irradiation-killed *Candida* species or *S. aureus*, cells were grown and washed as described above and then exposed in a thin liquid suspension to 4 doses of radiation (100 mJ/cm^2^) in a UV Stratalinker. Total killing was confirmed by plating 100-µl culture aliquots of UV-treated *Candida* (YPD agar) or *S. aureus* (TSA) and observing growth after incubation for 24 h at 30°C.

### Murine model of fungal-bacterial intra-abdominal infection. (i) Primary challenge.

Groups (*n* = 10) of 6-week-old outbred Swiss Webster or inbred C57BL/6J and RAG-1 KO mice were injected i.p. with various *Candida* species (1.75 × 10^7^/mouse) alone or in combination with *S. aureus* (8 × 10^7^/mouse), live or killed, in a volume of 200 µl at 14 to 60 days prior to rechallenge.

### (ii) Rechallenge.

For rechallenge, mice were injected i.p. with a lethal challenge of *C. albicans*, *C. krusei*, or *C. tropicalis* (1.75 × 10^7^/mouse) and *S. aureus* (8 × 10^7^/mouse) in a volume of 200 µl and observed for morbidity (hunched posture, inactivity, ruffled fur) and mortality up to 10 days after rechallenge. In some experiments, a subset of mice was sacrificed at earlier time points (4, 24, or 96 h) and peritoneal lavage fluid was collected for cellular analyses. For this, peritoneal cavities were injected with 2 ml of sterile saline followed by gentle massage of the peritoneal cavity. Peritoneal lavage fluid was then removed using a pipette inserted into a small incision in the abdominal cavity.

### (iii) Macrophage depletion.

Liposome-encapsulated clodronate and liposome vehicle (1 mg/mouse; Encapsula NanoSciences) were injected i.p. in 200 µl 1 day prior to rechallenge of animals with *C. albicans* and *S. aureus*. Depletion was confirmed by flow cytometry.

### (iv) Neutrophil depletion.

Mice were injected i.p. with either 200 μg rat anti-mouse Gr-1 (Ly6G/Ly6C) or rat IgG2A isotype control antibodies (Bio-X-Cell) in 200 µl sterile PBS to systemically deplete PMNLs 48 h prior to and 2 h after rechallenge with *C. albicans* and *S. aureus*. Injections were given every 2 days for the duration of the study. Depletion was confirmed by flow cytometry.

### (v) Flow cytometry.

Cells isolated from peritoneal lavage fluid collected at the time of rechallenge (separate mice) and at 4, 24, and 96 h after rechallenge (2 mice/group) were incubated with fluorophore-conjugated anti-CD45 (leucocyte common antigen), anti-Ly6G/C (PMNLs), anti-F4/80 (macrophages), anti-CD3 (T cells), and isotype control antibodies (BD Biosciences). Unstained cells and cells stained with individual fluorophores were used as compensation controls. Expression was analyzed using the BD Accuri C6 Plus flow cytometer (BD Biosciences) and FlowJo software.

### CFU analysis.

Microbial burdens in peritoneal lavage fluid were enumerated by serial dilution plating onto YPD agar containing 20 μg/ml nafcillin and 2 μg/ml vancomycin (for *C. albicans* enumeration) and TSA containing 20 μg/ml nafcillin and 2.5 μg/ml amphotericin B (for *S. aureus* enumeration) via the drop plate method (41). Plates were incubated overnight at 37°C. All CFU counts were expressed as the number of CFU per milliliter of peritoneal lavage fluid.

### Histological analysis.

Cytological smears prepared from peritoneal lavage fluid were spray-fixed with CytoPrep (Fisher) and stained with H&E by using the Protocol Hema 3 Stat pack (Fisher) for visualization of neutrophils. Smears on slides were visualized by standard light microscopy.

### Statistics.

Survival curves were compared using the log rank (Mantel-Cox) test. Significant differences were defined at a *P* level of <0.05. These statistical analyses were performed using Prism software (Graph Pad).
