# Supplementary figures and images for: Immune Protection against Lethal Fungal-Bacterial Intra-Abdominal Infections
Source: mBio. 2018 Jan 16;9(1):e01472-17. doi: 10.1128/mBio.01472-17 (PMC5770546; doi:10.1128/mBio.01472-17)

**Fig S1A**

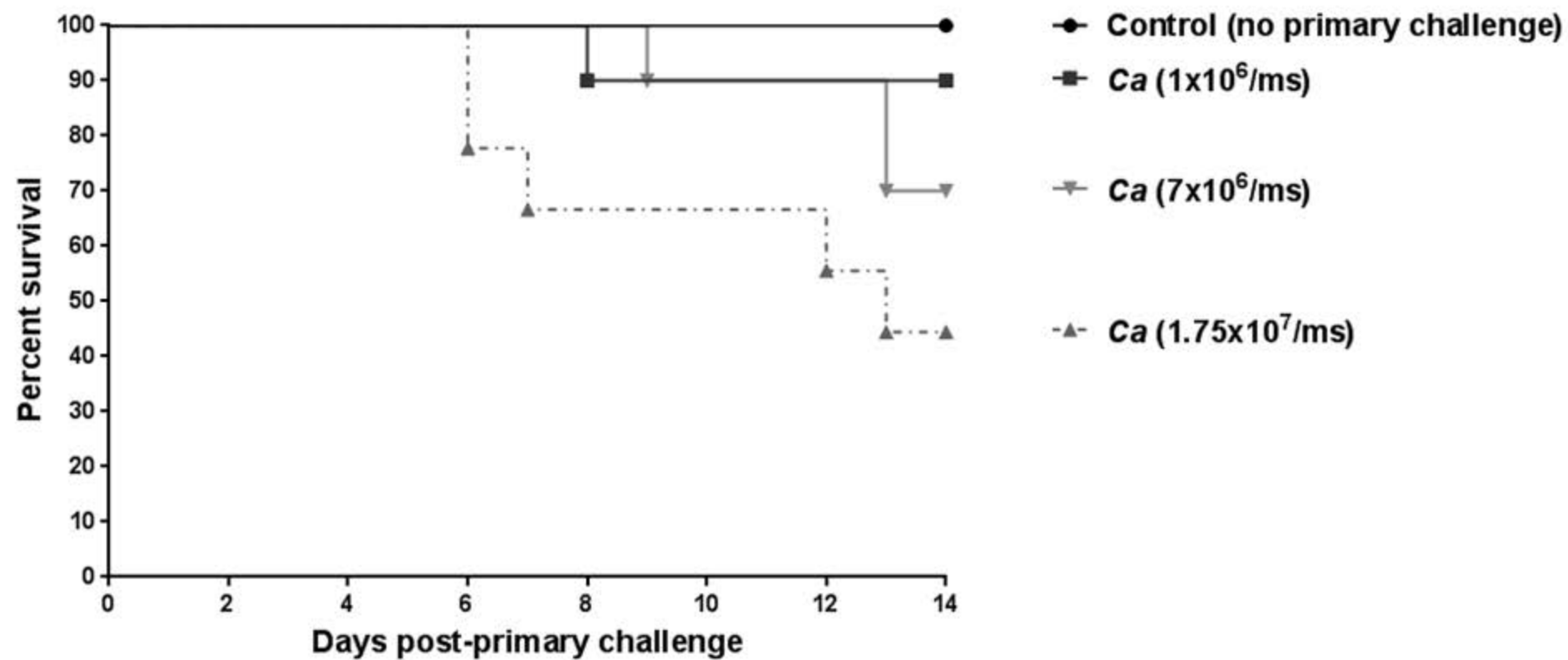

**Fig S1B**

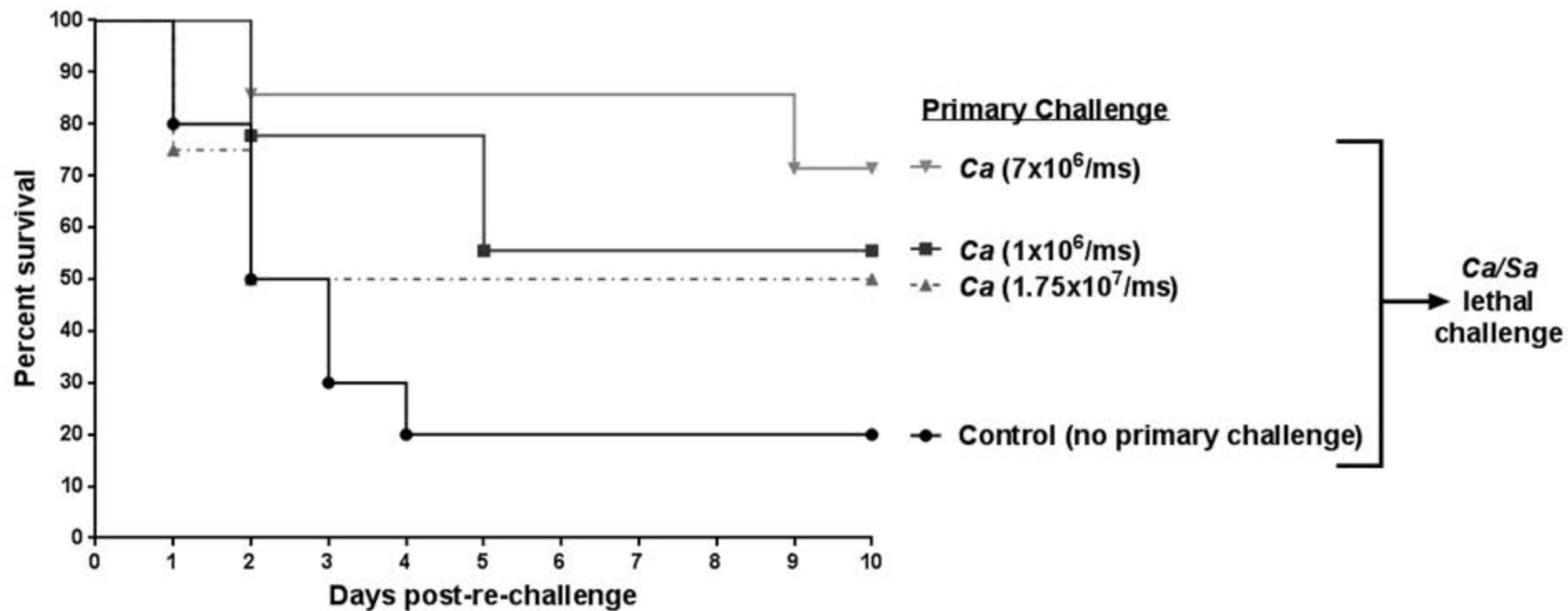

Supplement: FIG S1 [file mbo001183667sf1.pdf]
