# Supplementary material for: Immune Protection against Lethal Fungal-Bacterial Intra-Abdominal Infections
Source: mBio. 2018 Jan 16;9(1):e01472-17. doi: 10.1128/mBio.01472-17 (PMC5770546; doi:10.1128/mBio.01472-17)
Supplement: FIG S2 [file mbo001183667sf2.pdf]

Fig S2A

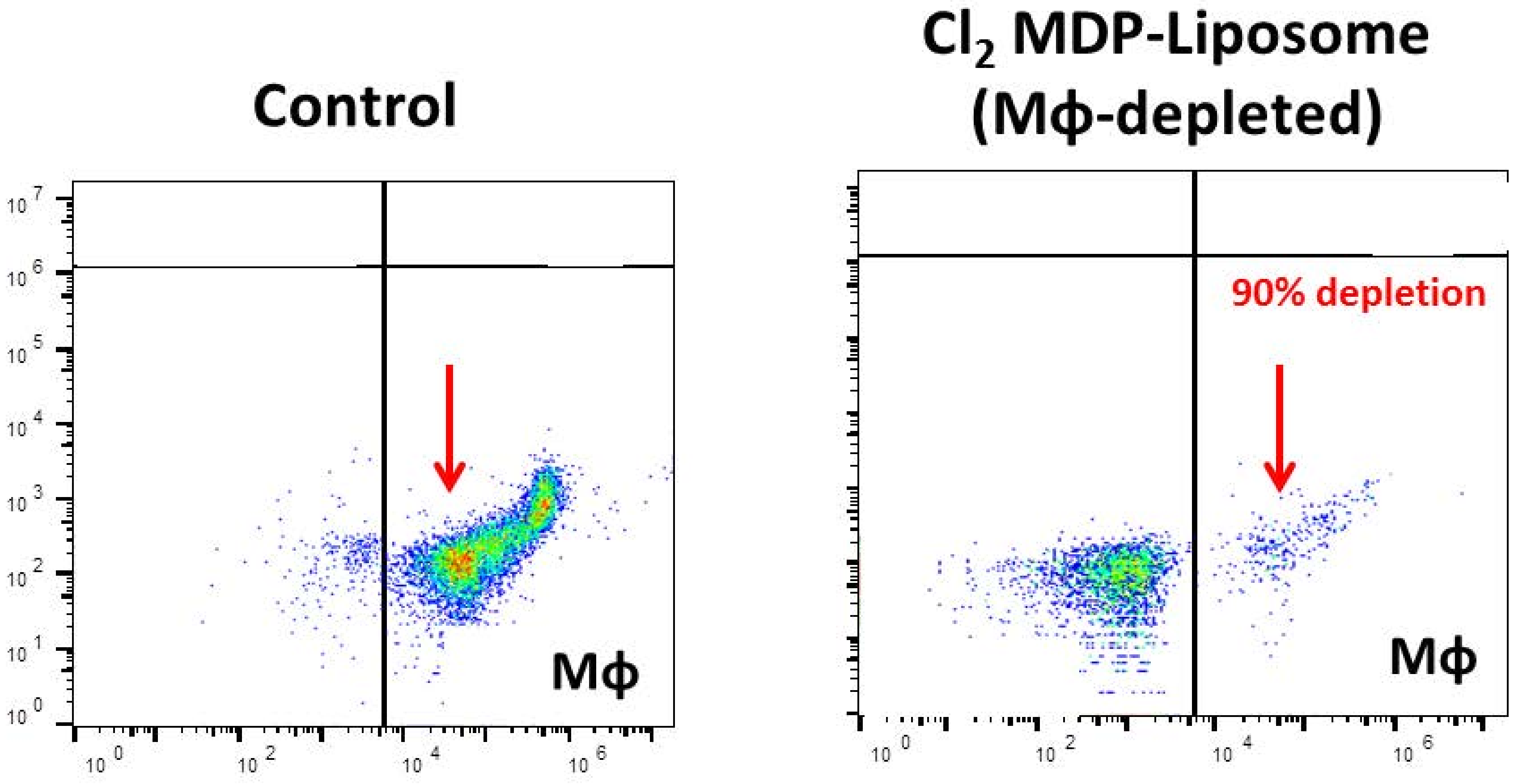
Control
Cl_2 MDP-Liposome
(Mϕ-depleted)
90% depletion
Mϕ
Mϕ
10 0
10 1
10 2
10 3
10 4
10 5
10 6
10 7

Fig S2B

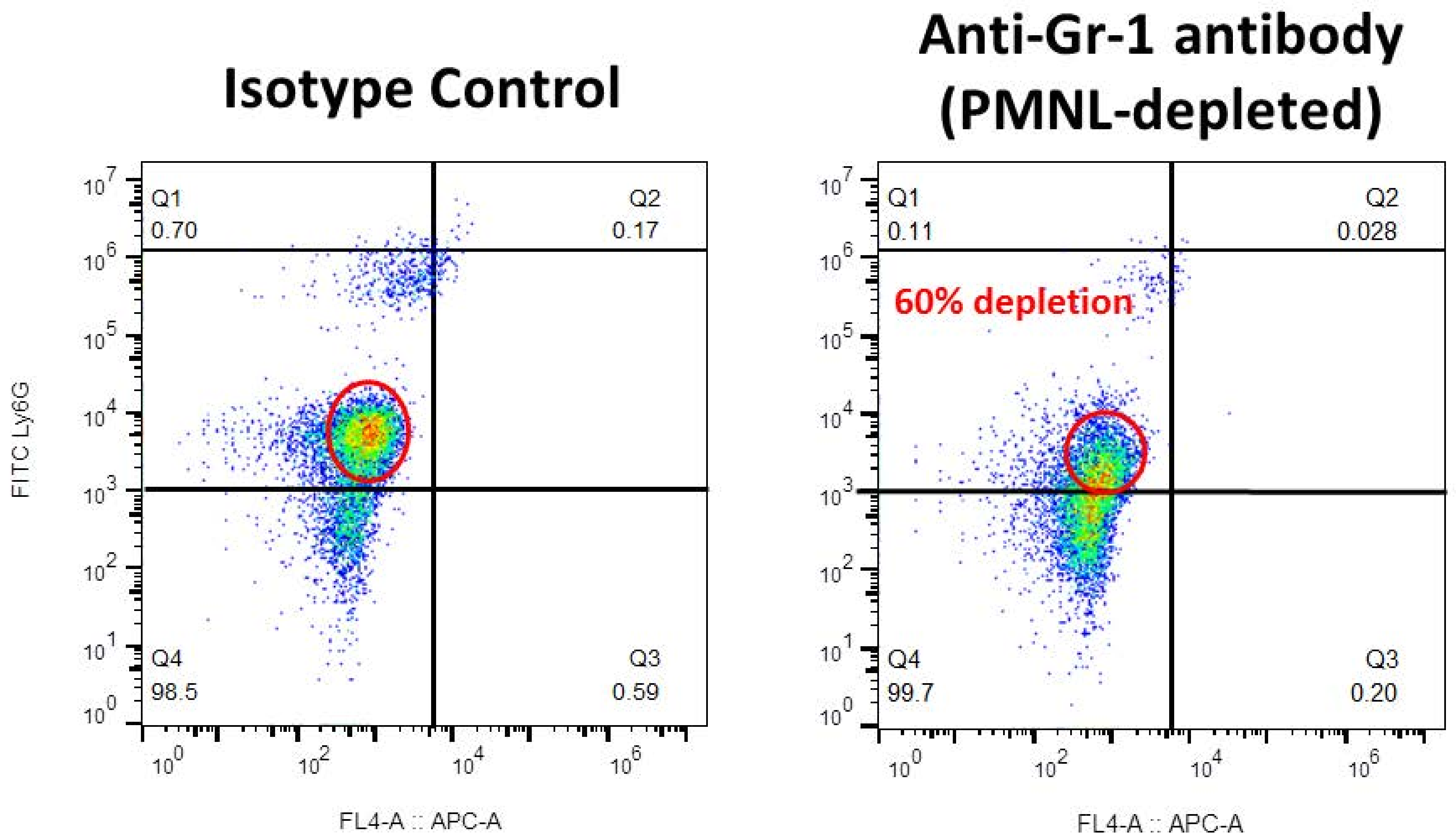
Isotype Control
Anti-Gr-1 antibody
(PMNL-depleted)
Q1
0.70
Q2
0.17
Q4
98.5
Q3
0.59
FITC Ly6G
FL4-A :: APC-A
Q1
0.11
Q2
0.028
60% depletion
Q4
99.7
Q3
0.20
FL4-A :: APC-A
